# A school-based physical activity intervention in primary school: effects on physical activity, sleep, aerobic fitness, and motor competence

**DOI:** 10.3389/fpubh.2024.1365782

**Published:** 2024-02-20

**Authors:** Júlio A. Costa, Susana Vale, Rita Cordovil, Luís P. Rodrigues, Vasco Cardoso, Rui Proença, Manuel Costa, Carlos Neto, João Brito, José Guilherme, André Seabra

**Affiliations:** ^1^Portugal Football School, Portuguese Football Federation, FPF, Oeiras, Portugal; ^2^Politécnico do Porto - Escola Superior de Educação, Porto, Portugal; ^3^CIAFEL/ITR - Universidade do Porto, Porto, Portugal; ^4^CIPER, Faculdade de Motricidade Humana, Universidade de Lisboa, Lisboa, Portugal; ^5^Faculdade de Motricidade Humana, Universidade de Lisboa, Lisboa, Portugal; ^6^Instituto Politécnico de Viana do Castelo, Escola Superior de Desporto e Lazer, SPRINT, Melgaço, Portugal; ^7^School D. Carlos I, Sintra, Portugal; ^8^Faculty of Sport, Centre of Research, Education, Innovation and Intervention in Sport, University of Porto, Porto, Portugal

**Keywords:** sedentarism, childhood, exercise, physical activity program, health

## Abstract

**Objective:**

The “Super Quinas” project evaluated the effectiveness of an intervention program to improve physical activity, aerobic fitness, sleep, and motor competence on children in primary school.

**Methods:**

The experimental group (*n* = 19) enrolled in a 12-week intervention program (one more extra-curricular activity class of 60 min per week) compared to the CG (*n* = 19), all aged 9–10 years. Physical activity (PA) and sleep were measured by accelerometry, and aerobic fitness was measured by Children’s Yo-Yo test (YYIR1C) during the 1st week (PRE), the 6th week (DUR), and the 12th week (POST) of the intervention program. Motor Competence in PRE and POST intervention was also assessed by the Motor Competence Assessment (MCA) instrument. Heart rate (HR, assessed using HR monitors), and enjoyment level were recorded during all intervention program classes. A linear mixed model analysis (i.e., within-subject analyses) was performed.

**Results:**

Comparing the EG and CG in DUR and POST, the EG spent ~18 min and ~ 34 min more time in moderate to vigorous physical activity (MVPA) per day (*p* < 0.001); had ~44 min and ~ 203 min less sedentary time per day (*p* < 0.001); performed more 44 and 128 m in the Children’s Yo-Yo test compared to CG (*p* < 0.001) and slept more 17 and 114 min per night (*p* < 0.001). In POST motor competence was significantly better (27%) in the EG compared to CG (*p* < 0.001). The %HRmax during the extra-curricular classes ranged between 65 and 81% (i.e., light to moderate intensities), and the enjoyment between fun and great fun.

**Conclusion:**

Our findings suggest that adding one more extra-curricular activity class of 60 min per week for 12 weeks effectively increased the levels of physical activity, aerobic fitness, sleep duration, and motor competence in children aged 9–10 years.

## 1 Introduction

Childhood physical activity is a global concern, with declining levels observed in many countries around the world (1). Portugal is no exception and has struggled with the challenge of declining physical activity levels among its youth (2). For instance, in Portugal overweight and obesity levels remain cautious, with one in three children in school-age are overweight (2, 3). Research from the latest round of the WHO European Childhood Obesity Surveillance Initiative carried out in 2018–2020 indicates that 29% of children aged 7–9 years in the participating countries were found to be living with overweight (including obesity – according to WHO definitions) (2, 3). This trend has raised significant public health concerns due to its profound impact on health and well-being of children. Research has consistently indicated that insufficient physical activity during childhood is associated with a range of adverse health outcomes, including obesity, cardiovascular disease, and metabolic disorders (4–7).

Given the critical importance of developing active lifestyles early in life, school-based interventions have emerged as a promising avenue for promoting physical activity (PA) among school-age children (8–10). These interventions often adopt multifaceted approaches, targeting not only PA levels, but also health related parameters, such as sleep, aerobic fitness, and motor competence (4, 10). Such comprehensive strategies recognize that these aspects of health are intertwined and can mutually influence each other (11, 12).

Aerobic fitness is a robust indicator of cardiovascular health and overall fitness, while motor competence, encompassing skills such as stability, locomotor and manipulative tasks, underpins a child’s ability to effectively engage in physical activities (13, 14). In fact, the development of motor competence is most crucial during childhood and school years, contributing to a solid foundation for a lifelong physical health (14, 15). School is the only shared environment for the entire age cohort of children and adolescents, hence offering the most powerful context to promote motor competence and physical fitness (16).

Additionally, sleep is emerging as a critical factor in the PA-health relationship (17). Inadequate sleep patterns have been linked to reduced PA levels in children (18, 19) while increased PA has been associated with improved sleep duration and quality, creating a reciprocal relationship between these two essential health behaviors (20, 21). In fact, an adequate amount of good sleep duration and efficiency is important for optimal health and functioning throughout life. Normal ranges for sleep duration in childhood have been published from several parts of the world (19); however, this has mostly been assessed by parental reports rather than objective measures. Moreover, within any population there is a wide range of sleep duration, and the potential consequences of reduced sleep have received little attention in community-based studies of children (18, 19). It is also important to note that, an association between reduced sleep and obesity in children has been noted (22, 23). However, little research has focused on what role environmental or behavioral factors such as daily exercise might play in the relationship between PA and sleep duration, especially in Portugal.

By examining these interconnected health parameters, we intent to get a comprehensive understanding of the effectiveness of a school-based PA intervention program in promoting overall health and well-being.

Thus, the aims of this study were to assess a school-based PA intervention program conducted on children in primary school, and to assess the impact of this intervention program on PA levels, sleep patterns, aerobic fitness, and motor competence. We hypothesized that adding one additional extra-curricular activity class of 60 min per week (i.e., 120 min of physical education per week in total), over a period of three months (i.e., 12 weeks), would significantly improve PA levels, sleep patterns, aerobic fitness, and motor competence on children in primary school.

## 2 Materials and methods

### 2.1 Participants and study design

The participants of this study were part of the pilot project “Super Quinas,” a 12-week intervention program focusing to increase daily PA levels, sleep patterns, aerobic fitness, and motor competence on children in primary school.

This pilot project was conducted in 44 schools across the country (i.e., in Portugal), and involved over 1,600 school-age children (6 to 10 years old). It took place between January and March 2023 at several school facilities, during extra-curricular activities and led by physical education teachers.

**Figure 1:**
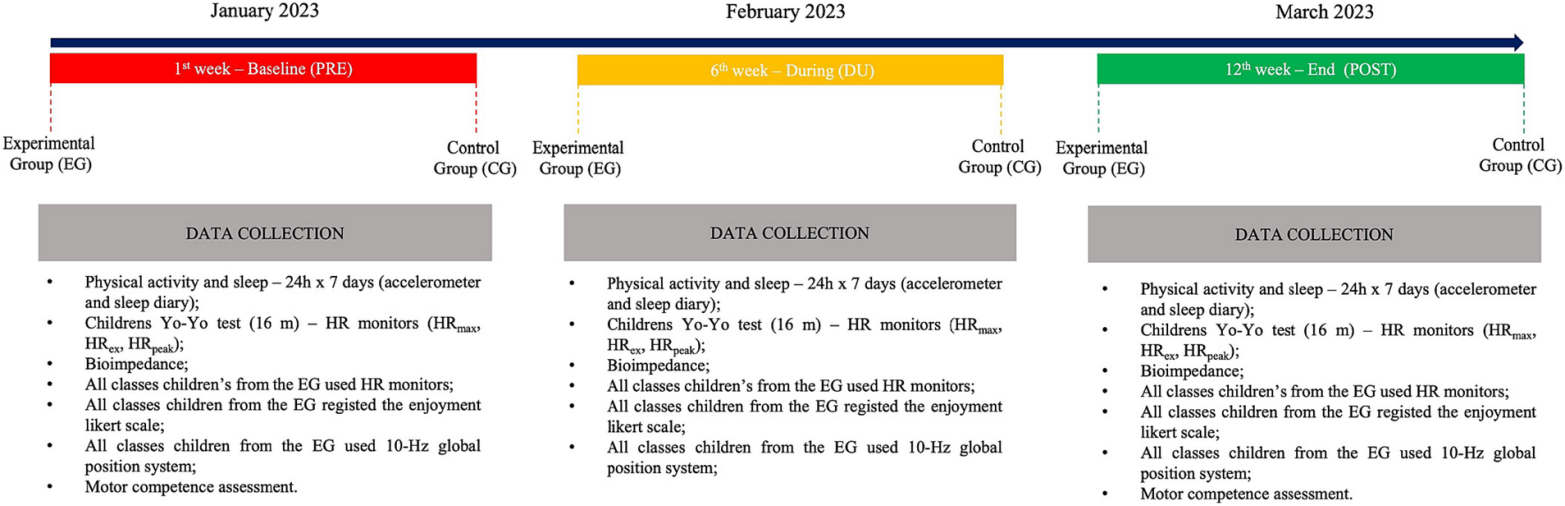
Study design of the “Super Quinas” physical intervention program in primary school.

All 1,600 children completed the MCA the PRE test in the 1st week and the POST test in the 12th week. Additionally, a sub-group of 38 children underwent more specific tests (i.e., PA, sleep, body composition and aerobic fitness) for a detailed analysis of the impact of this intervention project (Figure 1).

The experimental group (EG) comprised 8 girls and 11 boys (*n* = 19), while the control group (CG) included 9 girls and 10 boys (*n* = 19), with a mean age of 9.1 years and belonging to the 4th grade primary school. This study was conducted in one of the 44 primary schools located in Sintra (Portugal), under the supervision of one member of the research group (J.A.C.) and one physical education teacher. The school was chosen for convenience due to location and facilitation of data collection and analysis. It was chosen children from the 4th grade due to the available and interested of their parents to participate in the sub-group analyses. The use of medication or the presence of a pathology or clinical condition in which PA is contraindicated were considered as exclusion criteria. No children were excluded by the exclusion criteria mentioned above.

Sample size calculations were performed *a priori* for within-subject analyses using the G*Power software version 3.1.9.6, considering an effect size 0.25, a statistical power of 0.95 at *p* < 0.05. A sample size of at least 14 in each group was required.

Therefore, the present study focused on the analysis of a specific subgroup of children.

The EG was enrolled in 12-week intervention program, which included 120 min of physical activities per week (comprising 60 min of their usual physical education lesson plus an additional 60 min from the “Super Quinas” intervention program). The CG maintained their regular schedule, involving a 60-min physical educational lesson, and 60-min of an extra-curricular activity class per week (i.e., languages, art, or other non-PA).

In the intervention (i.e., for the EG), each 60-min session was organized into three parts related to different individual and group pre-sports games as described below:

1. The first part (~20 min) primarily focused on individual work emphasizing general motor skills. The goal was to promote body awareness through tasks like running, jumping, balancing, crawling, and climbing. Additionally, it aimed to enhance the body and ball relationship, incorporating ball manipulations, dribbling different types of balls, controlling the ball, and throwing or kicking at fixed and moving targets;
2. The second part (~30 min) centered on collaborative work in pairs or small groups. It aimed to further promote body awareness and the relationship with the ball;
3. The third part (~10 min) concentrated on group activities with all the group and included pre-sports games and small sided games that combined various actions such as passing, reception, dribbling, shooting, offense, or defense.

Weekly PA, sleep and aerobic fitness were measured, in January (PRE; 1st week), in February (DUR; 6th week) and in March (POST; 12th week) for the EG and CG. Motor competence was measured in PRE and POST, for the EG and CG. Heart rate (HR), external load, and sessions enjoyment level (1, *no fun* to 5, *great fun*) were recorded in all extra-curricular activity classes (i.e., 12 sessions in total) of the intervention program for the EG.

Prior to data collection, participants and their parents/legal guardians were informed about the study details (purpose, duration, type of intervention, risks and benefits). In fact, it is important to mention that in case of any injury or unexpected outcomes/results, the parents/legal guardians would be informed to guide their children to their respective family doctors/pediatricians, as indicated in the approved ethical document. They were also informed that they could withdraw from the study at any time, without any consequences. No child dropped out of the study. Written informed consent was obtained from the participants’ legal representatives and verbal assent was obtained from the children. The study was approved by the Ethical Committee of the Portugal Football School, Portuguese Football Federation (nr. CEPFS 17.2022).

### 2.2 Variables and measuring instruments

#### 2.2.1 Body composition

Body composition was assessed using an InBody 270 bio-impedance scale with an 8 Electrode Tetrapolar Electrode System with frequencies of 20 and 100 kHz. This allowed for the measurement of weight, body fat mass (%) and body mass index (BMI) (24). A portable stadiometer (Seca 213, Germany) was used to measure height. All measurements were conducted with participants lightly dressed (wearing only underwear and t-shirt) and barefoot.

#### 2.2.2 Physical activity and sleep

To estimate daily PA a tri-axial accelerometer (ActiGraph, model GT3X, Acticorp Co., Pensacola, FL, United States) was used at baseline (i.e., PRE), at the middle of the intervention program (i.e., DUR) and at the end of the study (i.e., POST). Participants wore the accelerometer during 7 consecutive days (i.e., Monday to Sunday) for each moment (i.e., at PRE, DUR, and POST).

A flexible elastic belt was securely fastened around the waist of each child. Children were asked to wear the accelerometer continuously for 24-h, with removal only during bathing, water-based activities, and in exceptional cases such as engaging in contact sports such as martial arts, where there was a risk of injury.

In the data analysis phase, records of PA performed on at least 4 days were considered, comprising 3 days during the week and 1 day over the weekend. Valid records needed a minimum of 8 h of recording per day (25, 26). Wear time validation was calculated using Troiano defaults (27, 28).

Accelerometer data files were collected according to the, respectively, cut point chosen to record the spontaneous and intermittent activities of children more accurately (25, 26). Evenson Children cut-points (25), validated cut-points recommended for children, were used to estimate time spent in *sedentary*, *light*, *moderate*, and *vigorous* intensity activity in children: *light* (101 to ≥2,295 counts per min), *moderate* (≥ 2,296 counts per min), and *vigorous* intensity (≥ 4,012 counts per min) PA (26, 29–31).

Sleep monitoring was assessed using the same accelerometers employed for PA measurements. However, during night sleep, participants were asked to wear the accelerometers on their non-dominant wrist (32, 33). Sleep variables were recorded every night over 7 consecutive days for each assessment point (i.e., at PRE, DUR and POST). Data was analyzed with the same software, using the Sadeh’s algorithm (34, 35). Sleep indices included sleep duration (amount of sleep in hours) and sleep efficiency (percentage of time in bed that was spent asleep) (35), which were analyzed according to the National Sleep Foundation (36, 37). A sleep duration <8 h was considered an indicator of inappropriate sleep duration, and a sleep efficiency ≤65% was considered an inappropriate sleep quality for children (36, 37).

#### 2.2.3 Aerobic fitness

The Children’s Yo-Yo test (YYIR1C) was measured during PRE (1st week), DUR (6th week), and POST (12th week) for the EG and CG. YYIR1C was developed for accounting for differences in running economy in children and has recently been reported to be a reliable and valid (construct validity) test for children’s of either sex (38, 39). This test uses the same acoustic progression, but shorter distance compared to Yo-Yo intermittent recovery test in its level 1 (YYIR1) version (38–40). Indeed, in the YYIR1C the children shuttle-run between two lines positioned 16 m apart, instead of 20 m, and walk over 4 m (instead of 5 m) in the 10s active recovery period (41, 42).

YYIR1C was performed at the school gym at the same time of the day, to account for circadian variation in human performance. All children were acquainted with the assessment procedures during dedicated physical education lessons in the week preceding the study.

#### 2.2.4 Motor competence assessment

Motor competence was assessed using the MCA test battery (15, 43), which comprises six tests, two for each component of motor competence: stability (lateral jumps and shifting platforms), locomotor (standing long jump and 10 m shuttle run), and manipulative (ball kicking velocity and ball throwing velocity). All tests are quantitative (product oriented), without a marked developmental (age) ceiling effect, and based on the child’s feasible execution of motor tasks.

In this study, testing conditions were arranged prior to beginning assessments, and children performed all tests in small groups (usually about five children per task). All participants completed a 10-min general and standardized warm-up before beginning the tests (14, 15). Examiners were previously trained in administering all tests, and the following requirements were standardized: (a) a proficient demonstration of each test technique was provided along with a verbal explanation; (b) every participant experimented with each task before the actual test administration; (c) the instructions emphasized that children should try to perform the task at their maximum capacity (e.g., “as fast as possible” for the stability tests and 4 ×10 shuttle run; “as far as possible” for the standing long jump; and “as hard as possible” for the manipulative tests); and (d) motivational, but no verbal feedback was provided (14, 15).

Results on each test of the three subscales were transformed into a percentile value by age and sex, according to the MCA norms (15). MCA subscales scores resulted from the average of the percentile values of the two constitutive tests, and the Total MCA score was calculated by the average of the subscales’ scores. This means that al MCA scores (tests, susbscales, and total MCA) represent percentile positions according to age and sex.

#### 2.2.5 Heart rate

HR data during extra-curricular activity classes for the EG was recorded in real time at 5 s intervals by short-range radio telemetry (Firstbeat Sports, Jyvaskyla, Finland). The monitors were attached to the children using an adjustable elastic chest strap. Data was transferred to a computer using the corporative software Firstbeat Sports Server version 4.7.3.1. HR_max_, which was considered as the highest value reached during the YYIR1C, served as the standard to establish the intensity zone of *moderate* to *very hard* at ≥70% HR_max_ (44). The software quantified the time spent at this intensity zone (i.e., ≥ 70% HR_max_) during physical education sessions, and data was presented as the percentage relative to the total training time. The software also calculated the percentage relative to the individual HR_max_ (%HR_max_) for the intensity zone as mentioned previously. The HR recording was not interrupted during the exercise transitions because the teacher organized the physical education sessions so that these breaks were not longer than 1 min.

#### 2.2.6 External load

Children used 10-Hz global position system (GPS) pods during extra-curricular activity classes (STATSports Apex, Northern Ireland) (45). In order to avoid inter-unit error, each child wore the same GPS unit throughout the data collection (46). Data were subsequently downloaded and adjusted to extra-curricular activity class exposure using corporate software (STATSports Apex, Northern Ireland). Total distance was used as an external load variable (47).

#### 2.2.7 Enjoyment level

Activities enjoyment was ascertained for the EG at the end of all extra-curricular activity class through a 5-points Likert scale (1, *nothing fun*; 2, *little fun*; 3, *indifferent*; 4, *fun*; 5, *very fun*). At the end of each class, each child was given a sheet with pictograms of human faces (i.e., emojis), which illustrate 5 levels of emotion (48). The child chose the one that most closely related to the enjoyment level felt during the proposed activities.

### 2.3 Statistical analyses

Sample distribution was tested using the Shapiro–Wilk test for PA, sleep, aerobic fitness, body composition and motor competence, for the EG and CG at PRE, DUR, and POST moments.

A linear mixed model analysis was performed to examine differences in *moderate* to *vigorous* PA (MVPA), sleep duration and efficiency indices, total distance performed in the aerobic fitness test and the total motor competence between the EG vs. CG at PRE, DUR and POST moments. A α-level of 0.05 was set as the level of significance for statistical comparisons. The PRE, DUR, and POST moments were included as a fixed effect and player identity (subject ID) as the random effect, between EG vs. CG.

Furthermore, among the recommended variance–covariance structure models, compound symmetry was selected according to the smallest Akaike Information Criterion assessment (49) based on the Maximum Likelihood method. Pairwise comparisons (Bonferroni) were used to show the mean differences for MVPA, sleep duration and efficiency, total distance performed in the aerobic fitness test and the total motor competence between EG vs. CG.

## 3 Results

Table 1 reports the anthropometric characteristics of the participants. No significant differences were found between EG vs. CG for all anthropometric measures (*p* > 0.05) in PRE moment. In the POST moment, children from the EG showed less weight, a lower fat mass percentage and a lower BMI compared to the CG (*p* ≤ 0.05).

**Table 1:** Participants’ anthropometric characteristics (*n* = 38).

	PRE (1st week)		POST (12th week)	
	EG (*n* = 19)	CG (*n* = 19)	EG (*n* = 19)	CG (*n* = 19)
Age (years)	9.1 (9–10)	9.1 (9–10)	9.2 (9–10)	9.2 (9–10)
Weight (kg)	33.1 (24.8–43.9)	33.2 (24.7–44.1)	27.3 (20.6–41.7)*#	36.9 (25–44.9)*
Height (m)	143 (132–156)	144 (134–158)	146 (138–158)	147 (136–159)
Body fat mass (%)	22 (12–41)	23 (12–39)	14 (11–21)*#	26 (12–44)*
BMI (kg/m^2^)	17.5 (15.1–24.3)	18.1 (13.4–22.6)	(11.3–20.7)*#	20.5 (16.8**–**24.7)*

Values are expressed in mean 95% confidence interval.

EG, experimental group; CG, control group; BMI, body max index.

* Significantly differences compared to PRE.

# Significantly differences compared to CG in POST.

**Figure 2:**
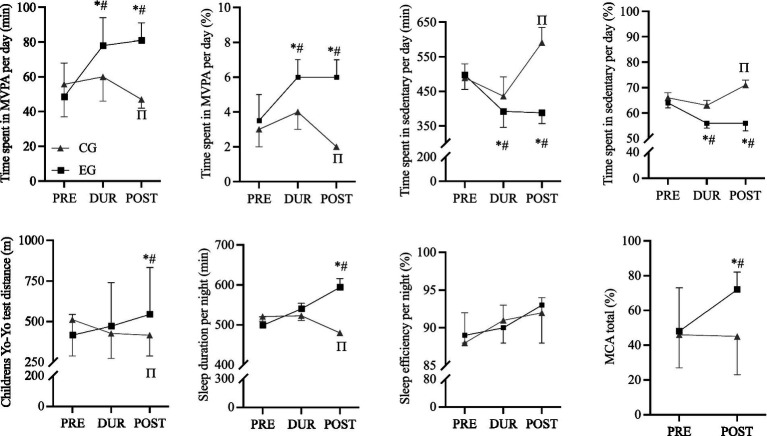
Descriptive data (Control group [CG] *n* = 19; Experimental group [EG] *n* = 19) responsiveness for time spent in *moderate* to *vigorous* physical activity (MVPA) per day; time spent in *sedentary* per day; Children’s Yo-Yo test (YYIR1C); sleep duration and sleep efficiency per night, and motor competence assessment (MCA) during PRE (1st week), DUR (6th week) and POST (12th week), in primary school children. Black lines (EG) and gray lines (CG) show group mean (95% confidence interval) during each evaluated moment PRE, DUR and POST. *Significantly different compared to PRE-EG. # Significantly different compared to POST-CG. Π Significantly different compared to PRE-CG.

There were no significant differences between groups (EG vs. CG) regarding PA, *sedentary* time, aerobic fitness, sleep duration and efficiency and motor competence, in PRE test (Figure 1). For the DUR and POST test moments, results show that EG spent more time in *moderate* to *vigorous* PA (MVPA) (DUR, more ~18 min; POST, more ~34 min) and less time in *sedentary* (DUR, less ~44 min; POST, less~203 min) per day, compared to the CG (*p* < 0.001). Children from the EG performed more 44 m and 128 m in the YYIR1C and slept more 17 and 114 min per night compared to CG (DUR (*p* < 0.05) and POST (*p* < 0.001), respectively) (Figure 2). No significant differences were found between EG vs. CG for sleep efficiency.

Regarding motor competence, significant differences were found between groups in POST test of evaluation. Motor competence was significantly better (i.e., more 27%) in the EG compared to CG (*p* < 0.001) (Figure 2).

When comparing PRE vs. POST test moments, results show that the CG decreased 1% (~ 9 min) of time in MVPA per day, spent 5% (~102 min) more time in *sedentary time* per day, performed less 96 m in the YYIR1C and slept less 41 min per night (*p* < 0.001) (Figure 2).

Regarding the EG, children increased by 3% (~33 min) in MVPA time per day, spent 8% (~110 min) less in *sedentary* time per day; performed more 128 m in the YYIR1C and slept more 95 min per night (*p* < 0.001) (Figure 2). No significant differences were found between EG vs. CG for sleep efficiency, in both groups (Figure 2).

Regarding MC, children from EG had significantly better motor competence scores (i.e., more 24%) in POST than in PRE (*p* < 0.001) (Figure 1). No significant differences were found for motor competence scores from CG between PRE and POST (*p* ≤ 0.05).

**Figure 3:**
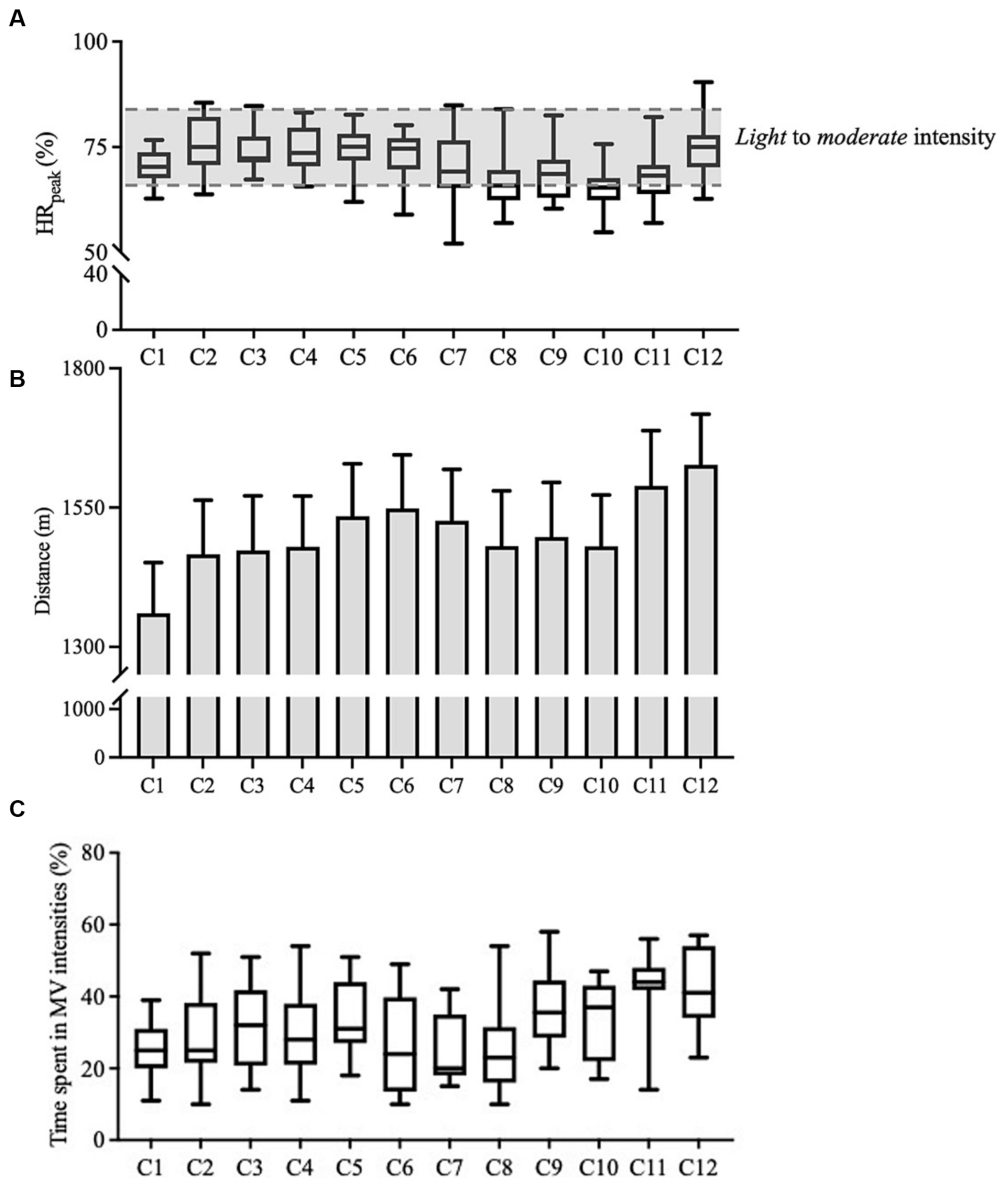
Descriptive data (Experimental group; *n* = 19) responsiveness for % heart max (%HR_max_), external load variables and the responsiveness for time spent in *moderate* to *very hard* (i.e., ≥ 70% of heart rate max) during 12-week intervention (one physical education session of 60 min per week), in primary school children. Graphic **(A–C)** Black lines show group mean (95% confidence interval) data. C, class; TD, total distance.

During the classes, the % HR_max_ ranged between 65 and 81% (i.e., *light* to *moderate* intensities) and total distance ranged between 1,360–1,627 m (Figure 3).

The time spent above 70% HR_max_ ranged between 15 and 60% (i.e., *moderate* to *very hard*).

Enjoyment ranged between 4 and 5 (i.e., *fun*–*great fun*). All children gave a 5 (*great fun*) in all extra-curricular activity classes with exception of one child that gave a 4 *(fun)* in the 4th extra-curricular activity class.

## 4 Discussion

The aim of this research was to present the findings of a school-based PA intervention program conducted among primary school children, assessing its impact on PA levels, sleep patterns, aerobic fitness, and MC. The comparison between the EG and CG at PRE and POST intervention moments revealed a significant and positive impact on several parameters, among primary school children, corroborating the study hypothesis. In general, the EG not only showed statistically significant improvements in weight, body fat mass, and body mass index but also exhibited positive changes in PA levels, aerobic fitness, and sleep duration, when compared to the CG. These improvements bring the EG closer to or in line with the WHO recommendations for children (50) in this specified age group (i.e., 9–10 years), indicating the effectiveness of the intervention program “Super Quinas.” These findings are also aligned with existing scientific literature that emphasizes the importance of PA interventions in improving overall health and well-being in children (4, 8, 10).

Regarding body composition, it is important to highlight that EG children showed significantly lower values of weight, body fat mass and body mass index in POST compared to PRE. This is in accordance with other school-based PA interventions: 11-week (51, 52), 12-week (53) and 6-month intervention (54). All these school-based PA studies have shown potential for yielding positive effects on body composition.

### 4.1 Physical activity, sleep, and aerobic fitness

It is well known that cardiovascular disease risk factors are associated with lower levels of PA and highly associated with a lower level of fitness in children (55). However, it is important to note that interventions focusing on PA within a school-based setting can potentially enhance both PA and fitness among healthy young individuals (16, 56). The most recent results from Portugal’s 2021 Report Card on Physical Activity for Children and Adolescents shows that less than 30% of children and adolescents achieve physical activity guidelines (2). In the present study, it was possible to find that the EG demonstrated a significant increase in MVPA compared to the CG, both at DUR and POST intervention moments. Moreover, the EG spent approximately 2% more time in MVPA per day at PRE, with this difference increasing to 4% in the POST intervention period. Accordingly, on *sedentary* time, the EG also exhibited substantial reductions compared to the CG, i.e., at PRE, the EG spent 7% less time in *sedentary* activities per day, which further increased to 15% in the POST period. This aligns with previous research indicating that structured exercise programs can effectively promote MVPA, contributing to improved cardiovascular fitness and overall health (57). Additionally, it also reinforces the importance of reducing sedentary behavior, as excessive sedentary has been linked to various health risks (58). In the current study, the EG improved the *moderate* to *vigorous* MVPA from PRE to POST intervention moments, approaching or reaching the WHO recommendations for children (50).

Regarding children’s sleep, children from the EG showed significantly more sleep duration at POST moment, with a significantly increase of 114 min, when compared to CG. The observed increase in sleep duration in the EG is consistent with research that suggests a bidirectional relationship between PA and sleep (17). Engaging in a regular PA can positively affect sleep duration by reducing anxiety and stress levels, promoting the release of endorphins, and contributing to better sleep patterns (59). This natural enhancement in sleep duration due to increased PA aligns with the findings of the current study. A study by Rhodes et al. (59) highlighted how PA can improve sleep quality and duration in children, indicating that an adequate sleep duration is essential for recovery and overall health, and exercise interventions have been shown to positively impact sleep quality and duration (17, 59).

It is also important to mention that PA and sleep could also be associated with the exercise intensity (60). In the present study the exercise intensity (monitored as a % HR_max_) revealed that the EG maintained intensities between 65 and 81% of HR_max_, indicating *light* to *moderate* intensities. This level of exercise intensity falls within the range typically recommended for promoting overall health and well-being in children’s (61). This level of exercise intensity encourages participation in a wider range of activities, contributing to an active and healthy lifestyle. The relationship between exercise intensity and sleep in children is also significant. The findings in the current study indicate that children in the EG experienced an increase in sleep duration following the intervention. The maintenance of *light* to *moderate* exercise intensities may have contributed to this positive effect on sleep. Research has shown that exercise at *moderate* intensities can enhance the overall quality and duration of sleep in children (62). For instance, Alnawwar et al. (62) have demonstrated that engaging in *moderate*-intensity physical exercise can result in improved sleep patterns, reducing the risk of sleep disturbances. Furthermore, *light-moderate* exercise intensities may help children fall asleep more easily and experience deeper, more restorative sleep (62).

Concerning the aerobic fitness, the YYIR1C test was significantly higher in the EG compared to CG at POST intervention revealing a better aerobic fitness after the 12-week intervention. This effect has been associated with a lower risk of cardiometabolic diseases, obesity, diabetes, and other health problems during the entire life cycle (63). Other studies have reported similar effects of intervention programs on aerobic fitness performance in children after 6 weeks (40), 11 weeks (52, 64) and 10 months (65).

### 4.2 Motor competence and enjoyment assessment

Motor competence is a crucial aspect of child development as it encompasses a range of fundamental movement skills that are essential for participating in PA and sports (14, 15). These skills include running, jumping, throwing, catching, and balance, among others. Motor competence is associated with a child’s overall physical development, coordination, and ability to engage in various physical activities. The findings of the present study showed that a school-based PA intervention had a positive impact on motor competence in primary school children. This is an important result because it aligns with the goals of physical education programs, which aim to improve children’s fundamental motor skills and physical literacy. Research supports the idea that well-designed PA interventions can enhance motor competence in children (20). A study conducted by Barnett et al. (4) found that structured PA programs in schools can significantly improve motor skills and fundamental movement abilities in children. In the current study, the EG displayed a 27% improvement in motor competence compared to the CG at POST intervention period. This emphasizes the effectiveness of exercise interventions in motor coordination, as found in the scientific literature (20, 66). Moreover, these results suggests that the intervention had a meaningful and positive impact on children’s motor skills. Such improvements in motor competence can have far-reaching benefits, not only in terms of PA and sports participation but also in promoting physical confidence and a lifelong interest in maintaining an active and healthy lifestyle (4, 67).

Finally, it is important to highlight children from the EG also reported high enjoyment levels, ranging from 4 to 5 on a scale of 1 (fun) to 5 (great fun). This suggests that the exercise program was fun and enjoyable, which is crucial for long-term adherence to PA programs (68).

Additionally, it is important to note that there are other factors that might influence PA levels, sleep patterns, aerobic fitness, and MC in children among primary school. Such as the cultural norms; socioeconomic disparities (e.g., access to recreational facilities and sleep environment); the educational system (e.g., school schedules and how the educational systems focus on promoting overall well-being); the technology and screen time (i.e., integration of technology in educational settings can influence sedentary behavior and impact both PA and sleep patterns); the parental involvement (e.g., modeling behaviors and sleep practices); the community and social support (e.g., community programs and social connections) and public health initiatives (e.g., government policies and accessibility of healthcare). Thu, all these factors are relevant for implementing and adapting the intervention, and tailored strategies would be necessary under certain social conditions.

## 5 Strengths and limitations

It is important to acknowledge the strengths and limitations of this study. It contributes to the existing knowledge by exploring the potential of a school-based intervention to improve the PA levels, sleep patterns, aerobic fitness, and motor competence among primary school children, which, to the best of our knowledge, has not been previously undertaken. Moreover, the use of objective instruments (e.g., accelerometers and HR monitors), further strengthened the design of our study. Overall, these strengths contribute to the robustness and credibility of our study’s findings.

Regarding the limitations, the sample size was small and not randomly assigned, which can increase the risk of bias. Activities outside school were not controlled in the analysis, nor the socioeconomic status or the presence of siblings in the family. Moreover, it is also very important to consider as limitation the fact that data were not analyzed according to the sex distribution (i.e., stratified analysis by sex) neither by location clusters.

## 6 Conclusion

Our findings indicate that adding one more physical education lesson of 60 min per week (i.e., a total of 120 min of physical education weekly), over three months (i.e., 12 weeks), resulted in significant improvements in PA levels, sleep patterns, aerobic fitness, and motor competence in children aged 9–10 years. Thus, this study highlights the role of the school-based programs as important determinants of PA levels in elementary schoolchildren. These data reinforce and justify that priority should be given to the development of national action programs that encourage the adoption of healthier lifestyles and to the creation of structural and environmental conditions favorable to children’s health.

## Data availability statement

The raw data supporting the conclusions of this article will be made available by the authors, without undue reservation.

## Ethics statement

The studies involving humans were approved by the Ethical Committee of the Portugal Football School, Portuguese Football Federation (nr. CEPFS 17.2022). The studies were conducted in accordance with the local legislation and institutional requirements. Written informed consent for participation in this study was provided by the participants' legal guardians/next of kin.

## Author contributions

JC: Conceptualization, Data curation, Formal analysis, Investigation, Methodology, Project administration, Resources, Software, Supervision, Validation, Visualization, Writing – original draft, Writing – review & editing. SV: Conceptualization, Data curation, Investigation, Methodology, Software, Writing – review & editing, Formal analysis. RC: Conceptualization, Data curation, Formal analysis, Investigation, Methodology, Software, Writing – review & editing. LR: Writing – review & editing, Investigation. VC: Investigation, Writing – review & editing. RP: Conceptualization, Visualization, Writing – review & editing, Data curation. MC: Conceptualization, Investigation, Validation, Visualization, Writing – review & editing, Supervision. CN: Conceptualization, Investigation, Supervision, Validation, Visualization, Writing – review & editing. JB: Conceptualization, Investigation, Validation, Visualization, Writing – review & editing, Supervision. JG: Conceptualization, Formal analysis, Investigation, Validation, Visualization, Writing – review & editing, Supervision. AS: Conceptualization, Data curation, Formal analysis, Investigation, Methodology, Project administration, Supervision, Validation, Visualization, Writing – original draft, Writing – review & editing, Funding acquisition, Resources.
